# The future of eating disorders research: an editorial

**DOI:** 10.1186/s40337-022-00538-5

**Published:** 2022-01-27

**Authors:** Stephen Touyz, Phillipa Hay

**Affiliations:** 1 InsideOut Institute, Charles Perkins Centre, University of Sydney, Camperdown, Australia; 2 Translational Health Research Institute, School of Medicine, Western Sydney University, Locked Bag 1797, Penrith South, NSW 2715 Australia

Much has changed since the first months of 2020 when we penned one of the first editorials to be published on COVID-19 and how it might impact on those with the lived experience of an eating disorder. At that time as Editors-in-Chief of the *Journal of Eating Disorders*, we instigated one of the first themed special issue entitled “Eating Disorders in the time of COVID-19 outbreak—Implications for now and the future”. The number of papers submitted and since published has exceeded all anticipations, demonstrating the attention that has been directed to this important topic and the emergence of systematic reviews [1]. There can now be no doubt that this pandemic has had a devastating impact on those with the lived experience of eating disorders with distress calls to the Butterfly Foundation phone hotline in Australia and similar access points around the globe soaring to a height of almost 200% in the UK [2]. We in no way want to detract from the urgency in ensuring that all of those experiencing distress from an eating disorder and wanting treatment, succeed in doing so despite the lockdowns and self-isolation imposed by health orders. On the other hand, however, the opportunities that COVID-19 has presented in terms of innovative health care delivery should be grasped at a time in our history when we have witnessed the greatest transformation in the innovation of advanced digital technologies. Such technology was responsible for the launch of the world’s first ever open access journal dedicated to eating disorders on the 22nd January 2013. It is hard to believe that a decade has elapsed since that time and that in 2022, the *Journal of Eating Disorders* will be in its10th year.

Looking over the topics of those publications that appeared in early years of this journal, there is clearly an ever greater need for research in our field. Those working in the field of family based treatments for adolescents with eating disorders particularly anorexia nervosa are exploring new avenues to develop better clinical efficacy such as multiple family therapy [3, 4], family based treatment (FBT) in the home, and combining FBT with dialectical behaviour and other therapies (see [5]). There has been precious little research to date that has transformed treatments for adults with eating disorders who are presenting much more of a challenge to those researching this field. In a recent *Lancet Psychiatry* meta-analyses of all the relevant randomised controlled trial research data on the role of cognitive behaviour therapy (CBT) in adult anorexia nervosa (AN), the authors concluded that in terms of outcome, it was not superior to treatment as usual [6]. Those venturing into novel areas for AN deserve our highest praise as it does take courage and fortitude to revisit theoretical understandings and venture into new avenues of neuro-modulation such as deep brain stimulation and rapid Transcranial Magnetic Stimulation and pharmacological agents such as ketamine and psilocybin [7, 8] when gold standards such as CBT prevail. This year also saw the opening of the first Australian residential centre for people with eating disorders which has embraced the concept of therapists with lived experience providing care [9]. Other areas are innovating. Those in our field who care for people with binge eating spectrum and comorbid metabolic disorders will be aware of the rapid rise of new glucagon-like peptide-1 receptor agonists used in combination with increasingly sophisticated behavioural weight loss therapies to treat type 2 diabetes. We cannot rest on our laurels.

Just published in the *Journal of Eating Disorders*, Levinson et al. [10] report on a proof of concept study and initial data in using networks to identify treatment targets for eating disorder treatment. They are adopting individualised network analyses utilising comprehensive longitudinal data via ecological momentary assessment to “model how dynamic systems of symptoms interrelate with each other to maintain pathology, within one person”. We need to truly embrace personalised care models for recovery and to learn what works for whom and when. It is no longer good enough to present data on attrition with the concept that people needed to stay with treatment and all would be well. Rather than people failing treatment, there is an imperative to refine the treatment to meet the person and their family’s needs. As enunciated by Gustafsson et al*.* [11] it is incumbent on us to increase our understanding of the treatment experience and to learn with those experiencing an eating disorder how to refine approaches and therapies.

We urgently need paradigm shifts to do for our field what has been done in developing vaccines and treatments for Covid-19. To mark the 10th year of the Journal of Eating Disorders, a series of special themed issues are planned. These include collections on medical assessment and management, environmental influences on eating disorders, disordered eating and body image, a trans-diagnostic understanding of binge eating, and a series of rapid reviews and updates for the field. It is hoped that the papers published in these as well as invited commentaries and editorials will be part of a global transformation of research to ultimately deliver more effective outcomes for people living with eating disorders. The researchers are here, as well as organisations such as the Academy of Eating Disorders and the Eating Disorders Research Society, other high calibre clinical/research bodies in many counties such as ANZAED (see the recent conference proceedings [12]) and a plethora of regular international and national conferences to rapidly disseminate research findings. If there was ever a time to feel optimistic about our field it is now.

## Data Availability

Not applicable.

## Abbreviations

AN: Anorexia Nervosa
ANZAED: Australian and New Zealand Academy for Eating Disorders
CBT: Cognitive Behaviour Therapy
COVID-19: Coronavirus disease
FBT: Family Based Treatment
